# Eyes on the enterprise: problematising the concept of a teaching-research nexus in UK higher education

**DOI:** 10.1007/s10734-020-00595-2

**Published:** 2020-08-03

**Authors:** Jim McKinley, Shona McIntosh, Lizzi Milligan, Agata Mikołajewska

**Affiliations:** 1 Institute of Education, University College London, WC1H 0AL, London, UK; 2 Department of Education, University of Bath, Bath, BA2 7AY UK

**Keywords:** Teaching-research nexus, Enterprise ideology, Problematising, UK higher education, Humanities and social sciences

## Abstract

Existing research into the relationship between teaching and research in higher education is mainly normative and atheoretical, resulting in assumptions of a close and beneficial connection between them. We problematise the idea of a *nexus* by undertaking a critical examination of the concept through the lens of educational ideologies to theorise the changes over time that shape the ways teaching and research are practised. Two hundred seven academic staff in the Humanities and Social Sciences were surveyed in 10 universities in England and Wales; the universities were identified as having strength in teaching, research, or in both. Along with analysis of interviews with senior managers at these universities, findings suggest that systemic forces which separate teaching and research are evident in institutional contexts with implications for the idea of a *nexus*. While the nexus may exist in theory, in practice, we argue that teaching and research can be pulled in different directions by institutional priorities. Furthermore, in institutions which adopt an enterprise ideology, there are signs of a nascent nexus emerging between research and innovation.

## Introduction

The concept of a ‘teaching-research nexus’ in higher education is one that is constantly in flux, yet persists as an academic ideal (Fanghanel et al. 2016). While argued that bringing cutting edge research into lectures and exposing students to the latest advances in the field in which they study are beneficial endeavours (Smeby 1986), more recently, the strength of this connection has been questioned in light of its relevance or value (Malcolm 2014) in the marketised era of higher education. A key criticism is a lack of empirical evidence about the characteristics of the nexus in practice (Elken and Wollscheid 2016; Hattie and Marsh 1996; Tight 2016). We approach the concept of the nexus with the understanding that its traditional and often unproblematised concept as a point of connection may, in fact, be insufficient to reflect the complexity of these two activities. Such complexity arises through the fragmentation of the higher education landscape, as research universities around the world gain increasing prestige in climbing global rankings (Marginson 2007), and in the UK post-1992, when universities’ research status increasingly became a proxy for quality (Boliver 2015). In addition, a historical lens is necessary to account for the way changes in higher education ideology affect how teaching and research are practised. We suggest that all these factors affect how the two activities relate to each other and, therefore, the concept of a nexus.

Currently, in the UK, academic staff in universities face pressures to meet the criteria for excellence in research, teaching, and most recently ‘knowledge’ through system-level evaluations, known as REF, TEF, and KEF, respectively, where performance is related to state funding. These macro-conditions separate teaching and research and, some argue, contribute to universities competing in a regime of excellence (Butler and Spoelstra 2014). Increasingly, the traditional coherence of institutional logic turns to competition (Shields and Watermeyer 2020) as staff are employed in positions that divide teaching and research in ways that are changing the nature of academic work (Macfarlane 2011; McIntosh et al. 2019). It can be argued that separating teaching and research at systemic and institutional levels brings increasing pressures on those attempting to maintain a connection between research and teaching in daily practice, a problem felt by universities globally. The question then is whether a teaching-research nexus can exist in UK higher education under these circumstances.

In this paper, we examine how conceptualisation of the nexus is influenced by ideologies of higher education (Trowler and Wareham 2007) via an online questionnaire with academic staff (*n* = 207) and interviews with senior managers (*n* = 11) in 10 universities in England and Wales. These included several teaching-oriented universities. To our knowledge, few studies, if any, involve universities that include those other than the research-strong/highest-ranked. We also explore the state of the nexus at a time when the ‘enterprise’ ideology, which is currently prevalent in the sector, may engender competition between teaching and research, and undermine the idealised complementary conceptualisation of the nexus.

## Literature review

While our study is focused on the UK, we draw on international literature, as the study is relevant to global contexts where teaching and research are competing. Globally, universities have been placing more emphasis on research, so we need to explore what impact this is having on academics and their practices. The UK has put in place quality assurance frameworks that have both instigated and addressed the imbalance. For the current study, it is therefore worthwhile to investigate the situation in the UK for comparative observations to be made internationally (Filippakou and Tapper 2007).

### The normative teaching-research nexus

Boyer’s (1990) identification of four separate but overlapping aspects of higher education work—discovery, integration, application, and teaching—introduces the notion of a link between knowledge production and teaching whereas ‘nexus’ (Jauch and Gentry 1976) is a term implying an immediacy between the teaching and research aspects of academic work. According to Tight (2016), the nexus emerged from a Humboldtian ideal of higher education. Dating from the nineteenth century, when scholars learned alongside masters that were experts in their field, this model of a symbiotic connection between teaching and research came to characterise the work of the European academic, reified in the standard academic contract in which teaching and research are equally apportioned. This gives rise to the view that a close coupling is fostered when there is a strong overlap between teaching and research in practice (Clark 1994).

However, such symbiosis rests on evocations of teaching and research as ideal types (Fanghanel et al. 2016), overlooking inherent dilemmas (Martin and Berry 1969) and fostering normative views about the nature of the nexus. Its existence was queried when no positive correlation was found between teaching and research (Hattie and Marsh 1996) leading to its being declared virtually dead soon after (Newby 1999). However, Healey and Jenkins (2009) note distinctions between research into student experiences of research and institutional practices to encourage a close connection between teaching and research. In doing so, they argue that the institution mediates the enactment of academic work in ways that can strengthen or weaken the relationship between teaching and research. The focus on student experiences of doing research, or learning from research-active faculty, described as ‘research-led teaching’ (e.g. Zamorski 2002) or ‘research-based teaching’ (e.g. Fuller et al. 2014) is distinct from concepts of a nexus in academic practice.

Such distinctions (teaching or research-led/research-based/research-informed) in discussions of the teaching-research nexus reflect scholars’ efforts to categorise different ‘dimensions’ of relationships between teaching and research. Krause (2007) identified seventeen such dimensions concerning a nexus on Australian university websites, and Trowler and Wareham (2008) developed a seven-dimension model based on a review of the nexus literature that subsumed Krause’s seventeen. This work suggests the identification of nexus models can provide useful frameworks for research and curricular policy decisions. However, the enactment of teaching and research is subject to influence from various factors that change over time. Following Becher and Trowler (2001), Trowler and Wareham (2007) point out that the relationship between teaching and research is profoundly influenced by academic disciplines, a point recently underscored by findings that pedagogies and curricula are valued differently across disciplines (Abbas et al. 2016).

Disciplines are also differently valued under different ideological conditions in higher education. The field of the Humanities and Social Sciences, for instance, is considered disadvantaged by commercially driven higher education priorities (Benneworth and Jongbloed 2010). Moreover, academic practice is influenced by global forces, and related trends such as the massification of higher education began in the USA after World War 2, which prompted a shift away from the Humboldtian-style nexus (Tight 2016). Additionally, teaching and research are practiced within a sector that places greater value on research than on teaching. Although, occasionally, teaching is the lead activity (Brew 2003; Fanghanel and Trowler 2008), studies on the nexus most often lead with research, describing a ‘research-teaching nexus’ (e.g. Farcas et al. 2017). This implicit, sector-wide value hierarchy (Bazeley 2010) may explain why the claims in many nexus studies rest on data collected in research-intensive universities (e.g. Cadez et al. 2017). This limitation is joined by a perceived lack of empirical evidence grounded in academics’ perceptions (Turk and Ledić 2016) that fail to recognise institutional variation (Jenkins and Healey 2005). Consequently, a recent review of existing research into the nexus concluded that, as a field, it is ‘ambiguous and sometimes contradictory’ (Elken and Wollscheid 2016: 3).

Uncritical idealisation of the nexus, therefore, evokes a normative, value-devoid concept that overlooks situated and historical influences. It also fails to recognise that its enactment unfolds under disciplinary, institutional, and systemic conditions that are shaped by overarching political ideologies. When resources such as time and money are scarce, understanding how teaching and research play out under different circumstances is crucial to discern whether the nexus is a help or a hindrance in conceptualising academic practice.

### Assumptions underpinning teaching and research in current academic practice

One task, then, is to critically examine the assumptions underpinning the existing research and question the normative stance that presents the nexus as ‘a good thing’. Another is to ask how it plays out in the current, highly marketised and globalised higher education sector (Marginson 2007) which is facing increasing financial uncertainty as a consequence of COVID-19. Even before Spring 2020, the funding of UK higher education was under strain, with universities competing for limited monetary resources while simultaneously attempting to meet rising expectations about standards of teaching and research from students, managers, and funders (Fanghanel et al. 2015). Indicators of success came to be understood in relation to performative measures of standards of excellence.

Some of these standards were enshrined in evaluative instruments which, notably, separate teaching and research. Currently, the Research Excellence Framework (REF), which is in its second iteration, has attracted criticism summarised by Butler and Spoelstra (2014) as contributing to a ‘regime’ of excellence that orientates researchers to work towards REF criteria. Meanwhile, the more recent Teaching Excellence Framework (TEF) acts as a proxy assessment of undergraduate teaching quality. The TEF has been criticised for being unlikely to achieve its stated aim of putting teaching at the heart of higher education (Forstenzer 2016) and of introducing Ofsted-style rankings likely to ‘punish institutions outside London and threaten arts and humanities courses’ (Fazackerley 2020: 1).

Separate evaluations of teaching and research at systemic level put pressure on the relationship between the two activities in daily practice (McIntosh et al. 2019). Academics, depending on the priorities of their institutions, may find the connection between teaching and research under strain (Macfarlane 2011) and be faced with daily compromise (McIntosh et al. 2019). The ideal of a symbiotic nexus is, therefore, undermined by structural separation of teaching and research. This separation raises the question about how and in what ways the nexus emerges in a marketised university sector, where teaching and research are evaluated, funded, and managed separately (Locke 2012).

However, and in addition to the limitations noted above, the field also:

… tends to be atheoretical. An empiricist ethic prevails, and underpinning this is a foundationalist ontological position, which assumes that a reality exists which can be apprehended by research which is sufficiently robust and extensive. An alternative position is a social constructionist one which stresses situational contingency. (Trowler and Wareham 2007: 2)

We meet this critique of the existing shortcomings of research in the field by recognising that any nexus is sensitive to the forces that shape teaching and research practices at the institutional level, as well as the wider landscape in which the institution operates. For this reason, it is important to theorise higher educational institutions as shaped by dominant ideologies.

### The nexus: in theory?

Conceptualising the nexus as an ongoing process (McKinley 2019) helps identify it as emerging with different characteristics under different structural conditions. We build on Jenkins and Healey’s (2005) argument that institutional-level strategies are one way in which the relationship between teaching and research is mediated, by contending that the perceptions academics have of these strategies must also be accounted for. This is because the organisational context and the culture within organisations cannot be assumed to support a close connection between teaching and research (Trowler and Wareham 2008). Organisations that emphasise research over teaching, for instance, bifurcate teaching and research at institutional level (Burke-Smalley et al. 2017), making it difficult for academics to meet the demands of an encroaching ideology of excellence (Butler and Spoelstra 2014) which manifest institutionally through managerialist adherence to criteria of systemic tools of evaluation, such as the TEF and REF.

Additionally, it is important to acknowledge that teaching and research will be variably interpreted in institutions, according to their different remits, histories, priorities, and values. In the UK, the Robbins Report (1963), for instance, categorised the ‘ancient’ universities separately from London, with its federation of colleges, the older civic universities separated again from teaching colleges and so forth. Differences in ‘types’ of higher education have also been categorised in accordance with their function (Moodie 2009; Tight 1996); and, under a ‘binary’ system of higher education (Scott 2014), teaching, rather than research, has been viewed as central to the post-1992 universities (MacFarlane and Hughes 2009). Where the institutional primary task is understood to be one or the other, the conditions for bringing both together will be more challenging than in institutions which value both equally and may jeopardise the existence of a nexus. However, publicly available information about university performance in the Universities Funding Council and The Times Good University Guide challenges ubiquitous university types based on, *inter alia*, age, location, research performance, and student intake (Lysons et al. 1998).

Although many university typologies are functional (Moodie 2009) and rest on assumptions of cohesion around institutions’ logics (Shields and Watermeyer 2020), we see this as over-simplistic, and agree with Trowler and Wareham (2007) who argue that academics’ practice in teaching and research are undertaken in relation to ideologies, that is, values, ideas, and beliefs that guide their working practices and shape the university as an institution. To discuss the nexus without recognising these shaping forces is to perpetuate the limitations in the field we noted above.

Trowler and Wareham (2007) illustrate changing attitudes and values towards teaching and research through a typology with four ideological perspectives on education. The typologies are arranged in a broadly chronological order, from oldest to most recent in time, with our own definitions provided for each in relation to a nexus:

- *Traditionalism*: close connection between researcher and research students; nexus strongly supported at postgraduate level
- *Progressivism*: connection enhanced when research activities encompass teaching; nexus enhanced when there is an overlap
- *Social reconstructionism*: close connection centering on social justice agenda; nexus is strong
- *Enterprise*: drift between teaching and research; transformation of the research/teaching nexus to the research/innovation nexus

We find that these are useful ways of conceptualising the systemic conditions under which teaching and research relate to each other and, thus, different potential enactments of a nexus. Our adaptation of this typology evokes the nexus as a lens through which to analyse higher education practices of teaching and research in relation to a shift towards enterprise as the new dominant educational ideology.

For example, in the traditional ideology, the Humboldtian ideal suggests a symbiotic nexus flourishes with the research student studying from the master advancing his field of scholarship, whereas this closeness under a progressive ideology that emphasises the transformation of individuals and knowledge will depend on the extent of overlap between teaching and research activities. Under social reconstructionism, the university is a place for developing human beings as critical, sceptical, and vigilant contributors to a more socially and politically just society (Abbas et al. 2016) and, when aligned with research objectives, can result in a strong nexus. However, in the enterprise ideology of the marketised university sector where higher education is positioned as an economic commodity (Naidoo 2003), what happens when the pairing of research and teaching has to accommodate further activities (Brennan et al. 2014) as evinced through the Knowledge Exchange Framework? Disciplinary differences further complicate the interplay of teaching and research under different ideologies. In the Humanities and the Social Sciences, for instance, moving away from social reconstructionism towards an enterprise ideology may prompt new alignments with innovation and entrepreneurship in ways that could compromise historic agendas of criticality and social justice and, consequently, the relationship between teaching and research. And finally, different universities will interpret and prioritise all these demands differently. The logical conclusion is that under differing ideologies, the teaching and research nexus unfolds in complex and institutionally variable ways.

In this paper, we contribute to debates about the teaching-research nexus in three ways. Firstly, we theorise the practices of teaching and research as related to educational ideologies by analysing academics’ perceptions of the relationship between teaching and research. Secondly, we analyse the nexus in relation to differing institutional priorities through data collected from a range of universities: institutions with strengths in teaching, strengths in research, and those where strengths in teaching and research are balanced. We do this analysis not according to Trowler and Wareham’s (2007) four ideologies, but instead use institutional differences (teaching-strong, research-strong, and balanced) to illustrate that there is evidence of a shift in these ideologies. Thirdly, we suggest that the teaching-research relationship is affected through variations in responses to the growing demands of an enterprise ideology. The original contribution lies not only in the new and extensive data set but also in extending the contentious debate about the characteristics, value, or even existence, of a nexus (Malcolm 2014; Tight 2016) once it is considered in relation to these wider historical and systemic contexts.

## Methodology

The study follows a concurrent, partially mixed design, with equal weight given to quantitative and qualitative data. Data were collected by two means: a survey of 207 practising academics in 10 UK universities via an online questionnaire and qualitative, semi-structured interviews with 11 senior managers at the same institutions.

### Sampling and sample

The sample was selected to focus on Humanities and Social Sciences faculty in universities across a spread of geographic locations. Through purposive sampling, we identified senior management in key positions such as pro-vice chancellors of teaching and/or research at universities in England and Wales with a range of institutional priorities. The Complete University Guide 2018 that uses TEF and REF scores served as an index to classify universities by their orientation: research-strong (*N* = 3), teaching-strong (*N* = 3), or balanced (*N* = 4). We understand that TEF and REF ratings are limited indicators (Forstenzer 2016), but following Moodie (2009), the Guide was considered useful in characterising universities in ways that extended dominant university typologies. Universities’ participation was further dependent on our ability to secure appropriate senior management participants within the project time frame. The questionnaire link was sent to academics working in the Humanities and Social Sciences at each university, distribution assisted by the participating managers. Questionnaire data were gathered from 207 participants (103 females, 87 males, 17 no specified gender). The majority of respondents were from balanced universities (*N* = 90), followed by academics from research-strong (*N* = 77) and teaching-strong (*N* = 40) universities (Table 1).

**Table 1:** Demographic information about participants by contract type and university type

	Teaching only contract	Both teaching and research	Research only contract	Total
Balanced	7	76	7	90
Research-strong	11	57	9	77
Teaching-strong	2	38	0	40
Total	20	171	16	207

### Materials

The semi-structured interview participants were well-established academics who had worked in Higher Education for 15–30 years and were, therefore, well-positioned to provide insights into teaching and research at their universities. Each participant was asked the same set of eight questions. Interviews lasted between 45 and 70 min and were conducted largely by telephone, audio-recorded, and transcribed before analysis, with the exception of one interview in which field notes were used as the participant chose not to have the interview recorded.

The survey was designed to investigate respondents’ perceived connections between teaching and research and identify factors related to any differences. The content of the survey was brainstormed by three of the researchers. For instance, we listed activities that we most commonly do in our own work as academics. This led to developing answer options for question number 3. We also provided space for open-ended responses to collect options not included in the list. When developing the survey questions, we could have used nexus categorisations along the lines of Krause (2007) or Trowler and Wareham (2008), as this could have yielded insights to the situation at a granular level. However, we elected not to use such categorisations, opting for a less clear identification of a nexus. Due to changes in such categorisations over time, we wanted to allow for the possibility that a nexus might not, in fact, exist for some respondents. While this may be a potential limitation to the study as a more comprehensive survey could have yielded more insights, the decision did allow us to draw our own conclusions based on university types. A draft survey was sent to an expert who suggested changes, which were applied to the final version of the survey. The questionnaire consisted of four parts:

- Background questions;
- Identifying teaching and research activities;
- Institutional priorities; and
- Motivation to work.

There were 19 questions, both open and closed, some with sub-questions to gain fuller responses. The first two parts are addressed in this paper. Survey results regarding the two latter parts were reported in McIntosh et al. (2019).

### Data analysis

The quantifiable data from the questionnaire, such as ‘contract type’ or research activities a participant engages in, were analysed using descriptive statistics to gain insights into participants’ perceptions of a teaching-research nexus as enacted in their institution, as well as how they understand the priorities towards teaching and research activities in their own work. The answers to open-ended questions (e.g. when participants are asked to explain how teaching and research overlap in their academic work) were analysed using thematic analysis to identify perspectives that aligned, or did not, with a nexus. The qualitative data gathered through the interviews were also analysed and coded using thematic analysis.

## Findings

Because we recognise the institutionally specific context matters in the way the nexus is perceived, in this paper, we focus on analysis of data that both provides insights into institutional differences and illuminates different demands that are placed on the teaching-research relationship.

### How do teaching and research overlap?

**Fig. 1:**
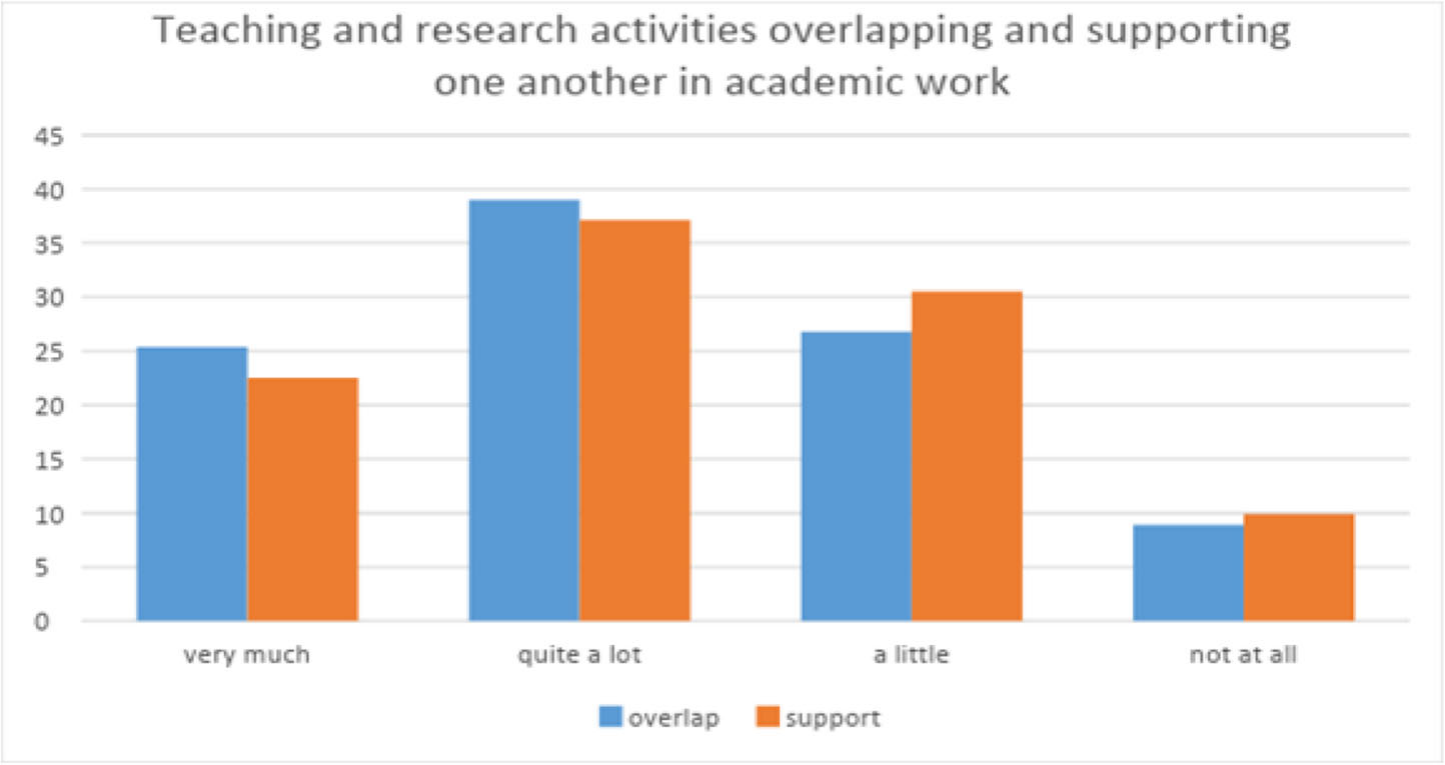
Perceived overlaps in teaching and research activities

Responses to teaching and research activities showed fragmented perceptions of an overlap (Fig. 1). Where some see the kind of complementarity inherent in the ideal nexus, there was a minority who reported little or no overlap or support.

Further detail about this fragmentation can be seen when activities are broken down (Table 3). Respondents were asked ‘Which of these do you consider to be *teaching* activities ( … )’, and which are ‘(...) *research* activities that you have undertaken in the 2017/18 academic year?’ As activities could be identified as both, answer options listed in Table 2 from 7 to 11 indicate overlapping classification. Supervision and keeping up to date with current research are clear examples of activities understood to be closely connected and, therefore, may be where a nexus might flourish. This is in keeping with the Humboldtian ideal under the traditional educational ideology.

**Table 2:** Academics’ perceived overlaps between teaching and research-related activities

No.	Activities	Teaching	Research
1.	Face to face teaching	198	
2.	Online teaching	81	
3.	Preparation	197	
4.	Designing new materials	191	
5.	Formative assessment	171	
6.	Summative assessment	185	
7.	Supervision of dissertations on taught programmes	170	188
8.	Supervision of postdoctoral research students	103	87
9.	Keeping up to date with current research	180	203
10.	Conference attendance	103	182
11.	Public engagement	97	133
12.	Developing new ideas		195
13.	Writing and publishing		195
14.	Working on unfunded research projects		166
15.	Working on funded research projects		141
16.	Applying for funded research projects		151

A nexus therefore exists as a close connection in a small number of specific activities. The activity with the highest number of responses, ‘keeping up to date with research’, which seems fundamental to an academic identity, is understood to be a holistic activity that is achieved via both teaching and research. This point is further supported by responses to open-ended questions:

Keeping up to date with current research - ensuring reading lists are updated so students are exposed to the latest debates/articles in the field. This also assists with research and engaging with recent debates. (balanced)

Another example of an activity that supports a nexus is the supervision of students. Most often, participants commented that supervising students is a stimulating and thought-provoking process mostly when considering postgraduate students:

Supervision of doctoral students ( … ) can be quite stimulating for ideas ( … ) marking students' work at postgrad and higher level as you learn from them. (balanced)

There was a strong emphasis on the exchange of knowledge between lecturers and students in the responses from all academics. However, only academics from teaching-strong universities expressed that they find support for their research from undergraduates:

I find if I am writing up my research, and try out the reporting of findings with my undergraduates, then it improves my articulation of my research in papers and reports. From time-to-time students do come up with questions or even insights that can inform my research, in particular shed light on analysis. (teaching-strong)

However, the clear identification of activities that are *only* research or *only* teaching (Table 2) raises a question. Is it the case that, in teaching-strong universities, there may be an emphasis on the first set of (teaching-focused) activities rather than the lower (research-focused) activities? The resulting institutional focus on teaching *or* research may serve to shape the activities undertaken by academics’ work. However, interview data show that collaboration of established academics and academics-soon-to-be exist in pockets, or as ‘examples’:

We’ve got applications for readerships where colleagues have published 20-30 papers in peer-reviewed journals, with their students, so there is, you know, really good examples of where the opportunities for students is fantastic. (teaching-strong)

Academics keeping up with current research and supervising research students, following a ‘traditionalist’ ideology and the Humboldtian ideal, as shown in the overlapping activities (Table 2), may very well perceive a nexus in their practice. However, academics whose workload tends towards teaching, or working on funded research, might struggle to identify a nexus. This supports our earlier point that the nexus must be understood within its contextual and historical context. It is also worth noting that public engagement, a relatively recent aspect of academic work, appears to be understood by many as connected to teaching and research, although elsewhere it is understood as closely connected with the innovation agenda (Pinheiro et al. 2015). Meanwhile, the development of new ideas being associated only with research may assume importance under an enterprise ideology, something that some universities may be adopting more readily than others.

### Institutional variation in the nexus

Institutional differences between perceptions of how teaching and research are supportive of each other, and how much they overlap, show broad differences in percentages when responses were collapsed into ‘very much/supportive’ or ‘not at all/unsupportive’ (Table 3). Analysis of potentially significant differences using the Kruskal-Wallis test on each item for the three groups (teaching-strong, research-strong, balanced) found no significant difference (*p* = 0.346) regarding the extent to which teaching and research support each other.

**Table 3:** Teaching and research support each other, by university type

	Teaching-strong (%)	Research-strong (%)	Balanced (%)
Very much/supportive	47	61	62
Not at all/unsupportive	53	39	38

However, a Kruskal-Wallis test for the item concerning perceived overlap between teaching and research found significant difference according to university type (*p* < 0.05). The results for teaching-strong universities were *p* < 0.02, SE = 10.83 while at balanced and research-strong universities, the result was *p* < 0.03, SE = 11.10. Pairwise comparisons of university type revealed that there was no significant difference between respondents at balanced and research-strong universities but participants in teaching-strong universities perceived *significantly less overlap* than those at balanced and research-strong universities. The conclusion is that, for academics in teaching-strong universities, teaching and research overlap much less.

Therefore, although there is an agreement that teaching and research are perceived to support each other, regardless of university type, differences emerge when respondents are specifically asked to consider how they overlap. In other words, an acceptance of a close connection may be strong in the abstract, but in practice, depends on the institutional type.

**Fig. 2:**
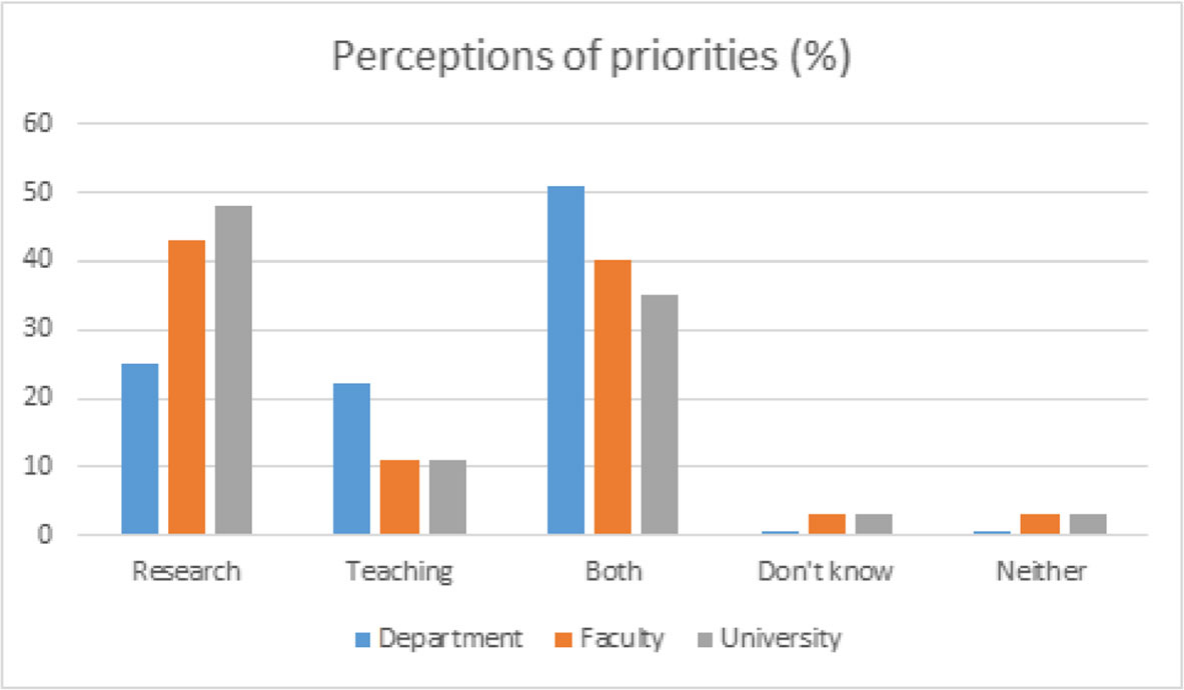
Perceived prioritisation of research, teaching, and both activities

We see further evidence to raise questions about the closeness of the connection in practice, and thus a possible drift toward an enterprise ideology, when examining different priorities at university, faculty, and departmental levels (Fig. 2).

Figure 2 shows that the further from practice, the more research is prioritised. The university is perceived to predominantly prioritise research, perhaps due to a stronger focus on enterprise, while at the department level, both teaching and research are perceived to be priorities. Teaching is perceived to be the lowest priority at all university levels, potentially undermining the complementarity required for a strong connection between teaching and research *in practice*.

In teaching-strong universities, we see evidence of pressure when demands of faculty and university differ:

Although research is increasingly prioritised at the faculty level ( … ), the university is still focused primarily on teaching. This means that I have to find time to conduct research outside of my teaching commitments. For example, it was impossible for me to pay the necessary attention to a research project I had funding for last term due to teaching commitments. I am now in a position where my teaching workload is lighter and I can try to catch up on the research I am conducting. (teaching-strong)

Similarly, in interviews with managers, teaching and research were separated structurally, reflecting their separation in the TEF and REF; for instance, ‘there’s a director of teaching and a director of research’ (balanced), or connection only very high up in the structures. However, one senior manager in a teaching-strong university disagreed with such structural separation:

For the first time the management of research and teaching was put kind of in the same person (...) it should be that every single department is underpinned by excellent teaching and research so it’s a very clear attempt to bring those two things together. (teaching-strong)

This comment points to the university’s drive to integrate quality research with teaching excellence. Indeed, for some managers, the nexus was the subject of intentional institutional policy:

There really is an expectation that all students have a really high-quality teaching experience and that they meet the professors as well as the junior lecturers; there is no kind of, you know, *some people are too important to teach*; all professors are expected to teach. (balanced)

This manager clearly sees benefits in students being taught by researchers and suggests a close connection between the two in institutional policy. Additionally, in sketching a pastiche of the distant professorial type, she recognises that some researchers may seek to escape teaching altogether; intentionally discouraging that expectation will shape resource-deployment decisions and priorities. For example, several teaching-focused initiatives were listed in this interview, including teaching pathway promotions, indicating an institutional commitment to balancing teaching and research. In such a context, it may be possible for a close connection between teaching and research to thrive.

However, this may differ across institution types. This was also evident in the questionnaire data; for example, when a university has ‘a lot of research-only people but it doesn’t have many teaching-only people’ (research-strong) whereas academics from balanced universities realise that they should be able to juggle the work associated with teaching and research activities and they are ‘very sceptical of either research-only or teaching-only contracts’ (balanced). The option for teaching and research balance in a work contract may be difficult in a teaching-focused university:

Teaching-only contracts makes it hard to catch up with the research demands and be able to move to a teaching and research contract (which has more prestige and security in my university). (teaching-strong)

Questionnaire respondents at research-strong universities also indicated awareness of this institutional difference:

I am fortunate that this faculty values teaching as well as research. My answers would be different in different universities, where I suspect teaching is undervalued relative to research. (research-strong)

Thus, where teaching is undervalued, the teaching-research nexus may struggle to flourish. However, there was evidence that some hope the TEF will enable a better institutional balance between teaching and research demands:

Academia is the only work where poor practice is rewarded. If you neglect teaching and students but write papers you will be rewarded. Conversely, being committed to students simply leads to more unrewarded work. This university is attempting to mitigate this by developing a teaching-related pathway. Until the political economy of the funding of HE changes this will always be the case (TEF may change this). (research-strong)

One respondent from a teaching-strong university who has been working in academia for several decades suggests there has been an increasing interest in strengthening the research profile of universities, regardless of their type:

Having taught at both pre and post 1992 institutions I can say with some authority that the teaching load is on a completely different scale at post-1992 institutions in terms of quantity. This has an impact on the time members of staff are able to devote to research and publications. While I have indicated above that teaching is prioritised and that is unlikely to change, there is no doubt that a research culture is developing at subject community, School and University levels. (teaching-strong)

The separation of teaching and research through systemic evaluative tools is seen to be shaping perceptions of a nexus for those managing as well as those undertaking academic work. However, research remains more highly valued.

Thus far, analysis shows an institutional variation in the way the nexus is perceived. Next, we examine the effects on the nexus under the introduction of innovation and its increasing intimacy with research.

### Institutional prioritisation (drifting toward enterprise)

The idea of a teaching-research nexus came under scrutiny from six of the interviewed managers, who questioned whether the nexus involved a close connection between teaching and research. They came from all three of the research-strong and two of the four balanced universities; none of those in teaching-strong universities was openly questioning the concept.

Maybe we have gone beyond it? But I was thinking that means we need to redefine that word, we need to, well revitalise it … making it something we actually physically recognise. (balanced)

Here, the interviewee recognises a disconnect between the *idea* of a nexus and seeing it in practice. Others were aware of the increasing incentives to partner research with industry:

[The university] Innovations Unit which is all about commercial dissemination of ideas; you know they're number one with industry for dissemination of research.”(research-strong)

Other examples of hitching research to enterprise were evident in interviews with managers in all three types of universities. Examples from research-strong institutions included university-based incubation and entrepreneurial schemes with prize money given to research with marketable potential that were open to undergraduates as well as postgraduates and academics. These strategies were found woven into institutions’ fabric, influencing teaching as well as research:

One of the core values of the university is enterprise, and I think we’re all aware that there’s quite a lot of challenges for [humanities and social sciences] in terms of speaking to the industrial strategy etc etc so we are being asked to write programmes that respond to that but they aren’t actually necessarily rooted in people’s research interest. (teaching-strong)

An explicit identification of enterprise as a core value is seen to drive teaching away from being research-driven and towards industrial goals, while the language of competition emerges as this manager describes the industrial strategy driving the direction of research:

The whole idea of the industrial strategy is about finding the edge to everything and how do we you know how do we find the next grapheme and how do we in a sense capture the quantum market. (balanced)

Industry, enterprise, and the marketability of ideas are evident, although this is not yet the case everywhere:

I feel lucky to have been working in an organisation which hasn’t radically gone down the line of splitting teaching and research but I am aware that a lot of organisations have to now quite a striking degree. (balanced)

A clear perception emerges that some ‘radical’ splitting of research from teaching is underway ‘now’ and the examples we share indicate that some universities are indeed drifting towards connecting research and innovation in pursuit of an enterprise agenda. This may be at the expense of the research teaching relationship, introducing further strain on the ‘ideal’ nexus.

These insights into varying institutional and wider systemic demands show teaching and research being pulled in different directions. And, when institutions are understood in relation to different educational ideologies, they can differently emphasise teaching and research. When research is harnessed to innovation, there is a drift towards an enterprise ideology that may undermine the connection between teaching and research and consequently weaken the nexus in institutions that explicitly pursue an enterprise ideology.

## Discussion and limitations

Our analysis contributes to debates in the field by raising questions about the teaching-research nexus in UK higher education today. While we find the idea of a nexus is persistent, one major criticism raised by Elken and Wollscheid (2016) is the partial nature of existing nexus research. It is often focused within individual practice or parts of institutions, with the result that claims are necessarily limited. The national scope of this study sought to avoid this limitation but, due to the disciplinary nuances attributed to teaching and research (Abbas et al. 2016; Becher and Trowler 2001), maintained some consistency by focusing on the Humanities and Social Sciences. Furthermore, we extended existing conceptualisations of university types beyond function that recognise institutional responses to historical shifts in the nature and conditions of higher education. We found variations between teaching and research, or research and innovation, may foster a disconnect between teaching and research exacerbated by an ideological shift towards enterprise.

The current study indicates that universities with strong research priorities can jeopardise a strong nexus. Therefore, Hattie and Marsh’s (1996) conclusion that teaching and research are, at best, loosely coupled may still hold where research is the institutional priority. Universities that adopt an enterprise ideology and are beginning to connect research with innovation may fall into this category, while universities who do not may still be motivated to foster a close connection between the two.

A limitation of the current study is the number of universities. While conducting the questionnaire in a larger number of universities could have potentially yielded more comprehensive insights, we kept the number to 10 for two reasons. First, the most important balance to maintain in the study was between the three university ‘types’, which we also tried to maintain with geographic diversity in the UK (initially, we contacted universities in Scotland and Northern Ireland as well, but none agreed to participate), *and* where there were willing senior managers to distribute the questionnaire and sit for the interview. Second, this was a funded project with a timeframe of just a few months, so pursuing further university connections was not possible. Furthermore, as the funder for the project specifically targets Humanities and Social Sciences, we only included universities where senior managers in these fields agreed to participate. A valuable extension of this study would be to conduct a refined version of the questionnaire across the UK.

## Implications and conclusion

We support Elken and Wollscheid (2016): 7) in asserting that the relationship between teaching and research is ‘a highly complex and multidimensional picture’. Furthermore, when they point out that existing research is inconclusive, they also identify an alignment with the traditional Humboldtian view of higher education, and one which appears increasingly dated. McKenzie et al. (2018): 1) claim that in Australian law schools, the nexus is a myth, driven from existence by cultures where research excellence frameworks create ‘an individualistic, competitive, disunited workplace’. In the UK today, the research excellence framework has been joined by two others, one focused on teaching and one on building links with industry. The academic who is operating under the evaluation of each of these different criteria of excellence may experience the different demands as an erosion of the quality of academic work (Butler and Spoelstra 2014). Research by Cadez et al. (2017) presents the results of a cross-discipline study of 620 academic staff in one research-focused Slovenian university. The university is characterised as ‘modern’ for its incentivisation of research output production, as opposed to the quality of the outputs or quality of teaching. Under such conditions, pressure to prioritise some activities over others may tip the balance to the extent that practice changes.

The adaptation of Trowler and Wareham’s (2007) earlier higher education typology to include the teaching-research nexus serves to emphasise the effect of changing ideologies on academic practice. It further highlights implications for the Humanities and Social Sciences, as well as providing yet more evidence that a shifting higher educational ideology towards marketisation and competition, and an ethic of neoliberal enterprise is changing the nature and purpose of the sector (Naidoo 2003; Marginson 2007; McIntosh et al. 2019). One recent example is the proposal by the UK government to measure the success of university courses by graduate earnings, entirely driven by this ideological shift (Fazackerley 2020). Equating quality with monetary earnings favours the disciplines leading to well-paid careers, such as medicine and law, over those in the Arts and Humanities sectors (see Benneworth and Jongbloed 2010 for stakeholder perspectives).

Such an initiative has further implications for the priorities within an institution and the position of disciplines within it. Under an enterprise ideology, the funding of disciplines may come to be increasingly closely linked to metrics promoting the characteristics of market enterprise over social reconstructionism in a way that will shape institutional priorities, destabilise the sector’s existing value hierarchy (Bazeley 2010), and rewrite the rules. Although it is not a new idea that students see their degree as a step towards employability (Tomlinson 2008), our analysis shows that these views emerge within institutions that adopt a particular ideology. The extent to which this will remain the case, however, is uncertain in the coming era of artificial intelligence, technological unemployment (Brown et al. 2018), and the COVID-19-fuelled economic crisis. This raises pressing questions about the purpose, nature, and material worth of higher education.

Since the enterprise perspective signals transformation of the nexus to align with innovation, and since innovation initiatives are used to build relationships between research and business, promoting them as social justice initiatives (as part of a social reconstruction ideology) might ultimately be economy-driven. Indeed, such a summation aligns with descriptions of a higher education sector in an increasingly fast-changing knowledge economy as ‘crucial … for the production, dissemination, and transfer of economically productive knowledge, innovation and technology’ (Naidoo 2003: 249). This is of particular relevance in research-strong and balanced universities that adopt enterprise values, where harnessing research to innovation serves to disconnect research from teaching.

From this disconnect, we see some significant implications concerning a teaching-research nexus. As universities respond to the incentives of the enterprise era, innovation, and especially innovation that is uncritically linked with student employability, is refocusing priority. Meanwhile, some institutional initiatives that intend to redress the disconnect, for example introducing teaching promotion pathways, may in fact be mirroring the systemic divide evident at national evaluation levels (Forstenzer 2016). Similarly, organisational structures such as departments for international education and innovation are sowing the seeds for the demise of the teaching-research nexus in ways that may reflect the changing character of higher education.

To close, we argue that while the teaching-research nexus may be thriving in the imagination of UK academics, in practice, it is fragile. Further research needs to attend to the nascent nexus in UK higher education: a ‘research-enterprise nexus’. As the enterprise ideology seeks to harness the economic potential of research, the value attributed to a teaching-research nexus is unclear. Future research on the concept of a nexus in higher education will need to consider these ideas alongside other current developments, such as the advent of artificial intelligence, perhaps in the context of the apparent identity crisis that higher education is currently experiencing.

## Data Availability

Will not be made available at this time
